# Importance–Performance Map Analysis of the Drivers for the Acceptance of Genetically Modified Food with a Theory of Planned Behavior Groundwork

**DOI:** 10.3390/foods14060932

**Published:** 2025-03-09

**Authors:** Jorge de Andrés-Sánchez, María Puelles-Gallo, Mar Souto-Romero, Mario Arias-Oliva

**Affiliations:** 1 Social and Business Research Lab, Universitat Rovira i Virgili, Campus de Bellissens, 43204 Reus, Spain; 2 Department of Marketing, Complutense University of Madrid, Campus de Somosaguas, s/n, Edif. 6, 28224 Pozuelo de Alarcón, Spain; mpuelles@ucm.es (M.P.-G.); mario.arias@ucm.es (M.A.-O.); 3 Department of Business Economics, Rey Juan Carlos University, Paseo de los Artilleros, 38, Vicálvaro, 28032 Madrid, Spain; mar.souto@urjc.es

**Keywords:** genetic modified food, genetic edited food, theory of planned behavior, importance–performance map analysis

## Abstract

The revolution in biotechnology at the end of the 20th century has improved agricultural productivity, reduced environmental impact, and enhanced food nutrition. However, genetically modified food (GMF) consumption remains low, particularly in the European Union, including Spain. This study develops a GMF acceptance model based on the Theory of Planned Behavior, incorporating five key variables alongside gender and age as control factors. Attitude-related variables include perceived benefit (PB) and perceived risk (PR). Perceived control factors involve food neophobia (PHOB) and knowledge (KNOWL) about GMFs. Additionally, subjective norm (SN) is considered. Using a sample of 728 Spanish consumers, the model was validated, explaining 70% of the variance and demonstrating strong predictive capability. The results so PB, KNOWL, and SN positively influence GMF acceptance, whereas PR and PHOB have negative effects. PB and SN emerged as the most influential variables, which are also highlighted as priorities in the importance–performance map analysis (IPMA). Based on these findings, strategies have been proposed to enhance perceived benefits and influence subjective norms, potentially increasing GMF acceptance. This study provides valuable insights into consumer behavior and offers guidance for policymakers and industry stakeholders to promote the adoption of GMFs.

## 1. Introduction

### 1.1. Genetic Modified Food

Ever since humans began developing agriculture and animal husbandry, they have devised methods to improve the genomes of plants and animals, laying the foundation for biotechnology [1]. By the late 20th century, a revolution in food technologies, driven by genetic advancements since the mid-century, led to new methods of food production, such as the genetic modification of plants and animals or cultured meat [1,2]. In the 21st century, food technology has become an active field of research because of its potential to address global challenges including climate change, animal protein shortages, food security, and sustainability [3].

Genetically modified foods (GMFs) are produced from genetically modified organisms (GMOs), which are living beings (i.e., plants, animals, or microorganisms) whose genetic material has been altered in ways that do not occur naturally through mating or recombination [4]. These modifications allow for the introduction of specific traits that are not naturally present in the original organism or the enhancement or suppression of existing traits [5]. Genetic modification offers several advantages such as increased resistance to pests, diseases, and extreme weather conditions, thereby improving crop yields and agricultural productivity. Additionally, it can enhance the nutritional profile of foods by intensifying essential nutrients such as vitamins and proteins. Genetic modifications also extend the shelf life of food products [6].

Thus, GMFs represent a potential solution to the 21st-century challenges. They help address food shortages by facilitating production and improving nutritional values. Additionally, they contribute to agricultural sustainability by reducing the need for pesticides, enabling cultivation under adverse climatic conditions, and reducing water consumption [7,8]. However, GMFs have raised concerns. Although studies conducted to date have not demonstrated significant adverse effects on human health [9], many consumers perceive risks such as unpredictable consequences of genetic modification, the potential creation of new toxins, or increased food allergenicity [10].

Environmental concerns appear to be more substantiated, including impacts on biodiversity, disruptions to the food chain, and genetic contamination [11]. Ideological concerns must also be considered, such as the potential monopolization of seeds by large corporations [12] and opposition from monotheistic religions, which view these biotechnological practices as intrusions into the divine order [13].

A distinction must be made between GMO production techniques that introduce external genes to create new traits in organisms, and those that modify existing genes. The first category includes transgenic foods, in which introduced genes come from sexually incompatible species, and cisgenic foods, in which genes originate from compatible species. The second category consists of gene editing, which aims to make precise and specific changes within the genome to achieve desired characteristics [14]. Although gene insertion produces results that are not possible through natural processes, gene editing creates organisms that already exist or could potentially be achieved using traditional agricultural and breeding techniques [5]. This distinction has led some authors to propose that gene-editing techniques should have a separate and less restrictive regulatory framework [15].

Despite the benefits associated with GMFs, opposition from public organizations and consumers remains widespread globally [16], with resistance being particularly strong in the European Union [17]. These perceptions can hinder development by discouraging investment in research on genetic modification technologies for food [18]. Thus far, the commercial presence of GMFs has been limited because they are unfamiliar to consumers and regulatory processes [18]. This is especially true in the European Union, where regulations of production and labeling are inefficient and inequitable for these products, thereby also discouraging producers and marketers [1].

### 1.2. Research Objectives and Theoretical Groundwork

Since the emergence of transgenic techniques in the early 1990s, advances in genome sequencing in the late 1990s and early 21st century have led to more precise methods known as gene-editing techniques, with CRISPR being the most notable [19]. Consequently, perceptions of GMFs have evolved over the past 30 years. In the European Union, strong opposition to food biotechnology, prevalent in the early 21st century, has gradually decreased, showing a slight shift toward approval [5]. This shift has been influenced by the greater recognition of biotechnology applications in other fields, such as medicine production [20]. Furthermore, when consumers understand the differences between older methods such as transgenesis and newer techniques like genome editing, they tend to view GMFs more favorably [21].

The considerations outlined above motivate our study, which analyzes the determinants of the intention to use (IU) GMFs among a sample of Spanish consumers in 2023, ranking these factors from highest to lowest priority. The goal is to assist potential GMF producers, marketers, and regulatory authorities in decision-making by identifying the key factors that should be prioritized to facilitate the wider acceptance of GMFs. The study of GMF is not new, even in Spain (see, for example, [22]). However, at the current stage of GMF evolution, most research focuses on North America and Asia, whereas studies on European Union consumers are scarce [18]. In the specific case of Spain, such studies are non-existent.

**Figure 1:**
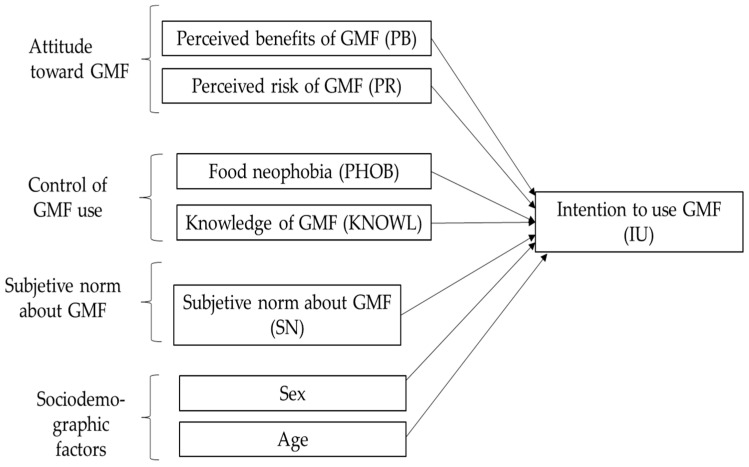
Theoretical TPB-based groundwork used in this paper.

The analysis of GMF acceptance requires a theoretical framework that allows a structured approach. This study is based on the Theory of Planned Behavior (TPB), which identifies three key factors explaining an individual’s intention to engage in a specific action: attitude, perceived control, and subjective norm [23]. This approach is widely used in studies on food-related decision-making [24] and is therefore commonly applied to the acceptance of novel foods, such as GMFs [3]. The proposed model is illustrated in Figure 1.

Attitude is defined as a person’s positive or negative evaluation of performing a specific behavior [23]. In the context of biotechnology-derived novel foods, both positive and negative aspects are commonly associated with health, price, and sustainability [25]. While positive outcomes are reflected in the latent variable of perceived benefits, negative ones are linked to perceived risks [26,27,28].

In TPB, perceived behavioral control refers to a person’s perception of their ability to perform a specific behavior; that is, the extent to which they believe they have control over the internal and external factors necessary to carry out that behavior [23]. The literature does not define this control precisely, as it can relate to both self-efficacy in performing an action and control over its outcomes [29]. In this study, we considered both aspects. As an internal factor related to the ability to consume GMFs, we include food neophobia, an irrational perception that discourages GMF consumption, even when consumers rationally acknowledge that they should accept it. In this case, food rejection is due to the process used in its production rather than the food itself, which is usually a common food [30]. The most relevant factor in controlling the outcomes of consuming a particular food is the level of knowledge, as a greater understanding allows for a better perception of its consequences. However, this knowledge cannot be perceived tangibly, because GMFs are substantially equivalent to conventional products, meaning that such consequences can only be understood through knowledge [31]. Food choices depend on multiple factors, such as the quality–price ratio or whether foods are healthier [32]. The perception of having an adequate assessment of these aspects depends on the knowledge of the ability of GMFs to provide such benefits.

The third aspect in the analysis of GMF acceptance is subjective norm, which refers to the perception of the degree of social pressure to perform a behavior [23]. In the context of GMFs, subjective norm factors include perceived societal beliefs about GMFs, whether they are supported by scientific evidence [33]. Therefore, this also encompasses moral, ethical, and religious perceptions of biotechnology within an individual’s social environment [13], as well as regulations concerning the commercialization and labeling of such products [5].

This study addressed two research objectives (ROs) based on the conceptual framework presented in Figure 1. These are differentiated as follows:

RO1 = To examine the capacity of the TPB-based model in Figure 1 to explain the acceptance of GMFs. The goal was to establish the explanatory and predictive power of the proposed model. It was evaluated using partial least squares structural equation modeling (PLS-SEM).

RO2 = To implement, in light of the model proposed in RO1, an importance–performance map analysis (IPMA) that allows for the visualization of which variables should receive special attention and improvement for the commercialization of GMFs. The importance–performance map (IPM) is constructed from two dimensions: the degree of importance of a variable in achieving an outcome, and the performance level attained in that variable [34]. This representation allows for the assessment of the need for intervention for each of the analyzed factors. For example, a factor with moderate relevance to acceptance but limited performance may require special attention because improving the performance of the driver could easily enhance the outcome level. Conversely, a driver of high importance with a high-performance level may not require further effort, as it is unlikely that significant improvements can be achieved beyond the attained level.

## 2. Hypothesis Development

### 2.1. Attitude Variables

#### 2.1.1. Perceived Benefit

Perceived benefit (PB) is one of the most relevant variables for explaining intention to use (IU) GMFs [35]. GMFs offer significant benefits in agricultural, environmental, and nutritional domains. One of the main advantages is the increase in agricultural productivity, as genetically modified crops can be designed to resist pests, diseases, and extreme weather conditions such as droughts or saline soils. This allows farmers to achieve higher yields with fewer resources, contributing to both food security in regions with agricultural limitations and availability of food at more affordable prices [36,37]. Additionally, the production of GMOs reduces the need for pesticides and herbicides, thereby reducing the environmental impact associated with the use of GMFs. GMFs can better adapt to the ongoing process of climate change, thereby increasing their resilience to global environmental transformations [33].

One of the most important factors in food consumption decisions is the nutritional properties [38]. In nutritional terms, GMFs can be designed to offer improvements in this regard. For example, varieties enriched with vitamins, minerals, and other essential nutrients can be developed. A clear example is so-called “golden rice”, which contains high levels of vitamin A and helps combat vitamin A deficiency in vulnerable communities [39]. GMFs can also be used in new pharmaceutical preparations and simultaneously to address global issues such as malnutrition [40].

The advantages related to price, health, and the environment have been shown to be significant for the acceptance of GMFs [1,41]. While the potential of these foods to improve health tends to be more relevant to perceived benefits than to environmental concerns [6] a lower price is considered more important than the two aforementioned factors [13,37].

The literature on the acceptance of new foods, and certainly those related to GMFs, identifies perceived benefit (PB) as one of the key variables for explaining their acceptance. This applies, for example, to the consumption of insects [42,43] and, of course, to the field of GMFs [22,44,45,46,47,48,49,50]. Thus, we propose:

**Hypothesis 1 (H1).**  *Perceived benefit is positively related to intention to use genetically modified food.*

#### 2.1.2. Perceived Risk

Perceived risk (PR) is one of the main drivers of negative consumer attitudes toward GMFs [26]. Although there is no scientific evidence that GMFs produce more negative effects than non-GMFs [9], people distrust genetic technologies due to their potential negative effects on health, such as allergies or toxicity, and on the environment [13]. It should also be acknowledged that there are no studies evaluating the long-term effects of GMF consumption [9], which leads to the perception among a significant portion of the population that there is some uncertainty within the scientific community about their effects [51].

One of the main myths regarding GMFs is that they can cause cancer [33], and the perception of that consequence is one of the main criteria considered by consumers when making decisions about food [38]. Zhao et al. [52] suggested that there may be an over-perception of risk regarding GMF consumption compared to other behaviors for which there is objectively more evidence of risk, such as smoking, workplace hazards, or food additives.

While some of the causes of the undesirable effects of GMFs can be theoretically argued in the case of transgenic and cisgenic products—for example, through horizontal gene transfer [9]—this reasoning does not apply to more modern food technologies like gene editing, as it does not involve the introduction of new genes and allows for the creation of products with compositions equivalent to existing ones [5].

Risk perception is negatively related to the intention to consume GMFs or, alternatively, that perceptions of safety encourage the use of such foods [22,36,44,45,46,48,53,54,55]. This also applies to other food innovations such as prepared dishes [56].

**Hypothesis 2 (H2).**  *The perception of higher risk decreases intention to use genetically modified foods.*

### 2.2. Variables Linked to Control

#### 2.2.1. Food Neophobia

Food neophobia (PHOB) is the reluctance to eat or avoid trying new or unfamiliar foods [57]. As with any phobia, it is an intense fear that is disproportionate to the actual danger, cannot be explained or reasoned away, is beyond voluntary control, and leads to avoidance of feared stimuli [58]. The biotechnology revolution that began in the late 20th century has enabled the creation of foods with numerous advantages; however, among the logical barriers to their adoption is PHOB [59].

When developing the concept of food neophobia, it is relevant to clarify what is meant by “new food”. In the context of GMF, this novelty does not come from the food itself, which is often indistinguishable from traditional food, but rather from the technology used to produce it [30]. Thus, some authors consider it more appropriate to refer to so-called food technology neophobia, which can be defined as consumers’ reluctance to try foods produced using novel technologies [60]. In the European Union, GMF must be explicitly labeled [16], which may generate phobia in individuals predisposed to consuming the non-genetically modified version of the food, even though the foods do not differ in appearance or taste from the non-genetically modified version [61].

It is a well-documented fact that foods labeled as natural or unprocessed are generally preferred by consumers [16,62]. Thus, the rejection of GMF by individuals with neophobia may be further stimulated by the fact that the more traditional a food production technology is perceived to be, the more natural it is seen [63]. It has been extensively reported that PHOB is a significant factor in the acceptance of foods. This applies to GMFs [37,50,64] and other novel food modalities, such as insects [43,65]. Therefore, we propose:

**Hypothesis 3 (H3).**  *Food neophobia is negatively related to intention to use genetically modified foods.*

#### 2.2.2. Knowledge of Genetic Modified Foods

Knowledge of GMFs (KNOWL) has been widely documented as relevant to understanding their acceptance [1,66]. This knowledge ranges from general knowledge of science and technology to more specific knowledge of genetics, biology, and biotechnology [66] or legal aspects such as certifications [67]. Although some knowledge that individuals possess may be objective and others subjective, based on perceptions [67], in the field of market research, it has been documented that the correlation between both types of knowledge is often positive [68].

The lack of information overestimates the perceived risk of GMFs and therefore hinders their acceptance [44,69]. When people understand how GMFs are developed, what benefits they offer, and what safety controls are in place, they tend to accept GMFs more. Thus, Refs. [21,51], in two similar experiments, showed that after informing a panel of potential consumers about the difference between older transgenesis techniques and more modern gene-editing techniques, consumers improved their perception of GMFs when they were produced using the more modern technique.

It is a widespread myth that more “natural” products are “safer”, while scientific evidence indicates that the safety of a food should be judged solely by its intrinsic components, regardless of the processes that led to its production [16,70]. Thus, consumer knowledge of institutions’ procedures and protocols that certify food safety increases their trust in them, and consequently, in the safety of certified foods and regulatory authorities [71]. Similarly, scientific studies supporting the safety of food products reinforce trust [51,55]. Thus, a positive attitude toward science has been shown to predispose individuals to a willingness to try GMFs produced through genome editing [53]. Thus, greater knowledge in the scientific literature on the consequences of GMFs is positively related to their acceptance [51]. This explains why various studies have shown that greater knowledge and awareness of GMFs positively influence their acceptance [36,37,45,61,72].

**Hypothesis 4 (H4).**  *Greater knowledge of genetically modified foods is positively related to acceptance.*

### 2.3. Subjective Norm

Subjective norm (SN) can be defined as the perception that there is societal pressure to indicate that a certain behavior is desirable [23]. Thus, if a person perceives that their close social circle supports or consumes a particular type of food, they are more likely to adopt a positive attitude toward it to align with the expectations of the group [73]. This influence is particularly pronounced among young individuals with clear references, such as friends, parents, or older siblings [74].

The relevance of SN is also more pronounced for foods such as GMFs, where consumers have limited knowledge of the technologies used to produce them and scientific evidence regarding their risks [75]. Individuals often follow social norms regarding food choices because they contain critical information about the safety, nutrition, or appropriateness of a particular food or way of eating [76]. In general, mainstream public opinion about GMFs, although it has varied over the years and seems to be increasingly favorable, tends to be more negative than positive, both in the European Union [20,77,78] and other geographic regions [79,80].

Additionally, ethical, economic, and environmental concerns contribute to negative social perceptions, which hinders the acceptance of GMFs [81]. Some people fear that GMFs disproportionately benefit large agricultural corporations, promoting seed monopolization and increasing costs for farmers [82]. There are also concerns about potential long-term effects on biodiversity, such as gene transfer to wild species or the development of resistance in pests and weeds [40].

In the context of insect consumption, Refs. [42,67,83] observed that subjective norms are a particularly relevant factor for acceptance. This has also been reported in street food [84], organic food [25], green food [85], and processed food [73] settings. In the GMF context, Refs. [27,28] also observed a positive impact of subjective norms on the willingness to try GMFs.

**Hypothesis 5 (H5).**  *Subjective norms positively influence acceptance of genetically modified foods.*

### 2.4. Sociodemographic Variables

In analyses of acceptance of new foods, it is common to include sociodemographic variables as control variables, with the most relevant being sex and age. As control variables, we introduce them in an exploratory manner without making any hypotheses about the direction of their relationship with the intention to consume GMFs.

The inclusion of sex is justified both by biological differences in the nutritional needs of females and males, and by cultural differences [86]. Men tend to show greater acceptance of new forms of food such as entomophagy [87], cultured meat [88], and GMFs [21,51,59,72]. However, there is also a wide range of studies that do not find a significant relationship between sex and acceptance of new foods [47,89,90].

Age can be a driver of food preferences. Makowska et al. [91] indicated that while members of Generation X and Baby Boomers tend to value food quality, Zoomers and Millennials focus more on price. On the other hand, Ref. [92] pointed out that while older generations prefer familiar foods, younger individuals consider sustainability and whether foods are healthier in their decisions. The findings in the field of new food technologies are contradictory. Although the mainstream trend may lean toward observing a negative relationship between age and acceptance of GMFs [59], there are also reports that suggest a more positive attitude among older individuals [52,72,89], or simply no significant relationship [51,55].

## 3. Materials and Methods

### 3.1. Sampling and Sample

This study employed a structured, self-administered online survey targeting individuals aged 18 years and older residing in Spain. It was conducted during March and April 2023 and was distributed among users of food opinion platforms using a non-probabilistic convenience sampling method. Although the results of convenience sampling are, in principle, only robust within that specific context, we believe that this sampling approach offers several advantages in our study [93]. The responses came from individuals who were genuinely motivated by the subject matter and possessed knowledge about food, even if they had not received formal education in the field. As a result, their responses were likely to be given careful consideration. Furthermore, it is very likely that these individuals’ judgments influence the eating habits of people close to them, who do not show as much interest in food and nutrition topics.

A total of 826 questionnaires were collected. However, we only considered those in which the items related to IU, PB, PR, PHOB, SN, and KNOWL were fully completed, ultimately analyzing 728 responses.

Table 1 presents respondents’ profiles. It can be observed that 38.46% were identified as male and 52.20% as female, and the remaining 9.34% either preferred not to answer or did not identify with a binary gender option. Regarding respondents’ age, 50.05% reported being 45 years old or younger, while 41.48% were 46 years or older. The average patient age was 47 years, with a standard deviation of 19.28 years. In terms of education, 3.02% reported having only primary education or less, 26.65% had completed only secondary education, and 61.68% had university education. Concerning income levels, 49.04% of the respondents stated that they had a monthly income below EUR 1750, whereas 22.66% reported having an income equal to or above this threshold.

The sample size was adequate in terms of the statistical power needed to assess the fit of a regression model, as shown in Figure 1, which suggests a model with seven regressors. Using the GPower 3.1 software [94], it was observed that significance tests for both individual coefficients and the overall model, considering a 5% significance level, provide 90% power for very small effect sizes (0.015).

### 3.2. Measurement of Variables

The first version of the questionnaire was reviewed by 11 university professors with diverse profiles related to food and GMFs, including researchers in agricultural product marketing, nutrition scholars, and biotechnology experts. Their recommendations did not lead to substantial changes in the questionnaire, but helped improve its readability.

The questionnaire began with a brief explanation of the concept of genetically modified food. Following this introduction, the respondents were asked to answer items measuring the factors involved in the explanatory model developed in the second section. The scales used to measure the dependent variable, intention to use (IU), and the explanatory variables, along with their sources, are shown in Table 2.

The items considered in this study are listed in Table 2. All questions were answered on a Likert scale ranging from 0 (“strongly disagree”) to 10 (“strongly agree”), with the intermediate position represented by response 5. Despite that this scale can be found excessive in respect to more common scales with less points, it has some good properties. Firstly, most individuals can perceive more nuances than what 4-, 5-, or 7-point scales allow. Secondly, this scale offers greater sensitivity and more closely aligns with an interval level of measurement and normality. Moreover, a 0-to-10 range is widely understood and easily interpreted by most people [95].

The control variables, gender and age, were modeled as dichotomous variables. For gender, 1 stands for women and 0 for men, while for age, 1 corresponds to individuals over 45 years old and 0 to those who reported being under 45 years.

Although the binary separation in the age range has a certain degree of arbitrariness, in our case, it has been chosen based on four reasons. The first is that this age approximately corresponds to the median age in the sample used in this paper. Secondly, following Ref. [96], it aligns with the division made by many authors between the phases of early maturity (30 to 45 years) and maturity (46 to 60 years) [96]. Finally, at the time of the survey, individuals aged 45 years or older could be associated with Generation X or Baby Boomers, while younger individuals would correspond to Millennials and Zoomers. Specifically, the cutoff age is set for those born in 1978, which falls within the range considered the diffuse boundary between Generation X and Millennials, generally placed from the mid-1970s to 1980 [96]. Finally, the results of studies such as [91] suggest that, on one hand, the food preferences of Generation X and Baby Boomers may be quite homogeneous, as is the case among Millennials and Zoomers. The gap in preferences appears to be between members of Millennials and Generation X.

**Table 2:** Items of the latent variables of the model developed in Section 2.

Intention to Use (IU): Adapted from [97] IU1: I intend to eat genetically modified foods. IU2: I predict that I will consume genetically modified foods.
Perceived Benefits (PB): Adapted from [49,98]. PB1: I find it useful to produce and consume genetically modified foods. PB2: The production and consumption of genetically modified foods allow me to achieve the goals that I consider important. PB3: The production and consumption of genetically modified foods allow me to achieve important goals more quickly. PB4: Producing and consuming genetically modified foods has many benefits.
Perceived Risk (PR): Adapted from [99]. PR1: Consuming genetically modified foods is risky. PR2: There is too much uncertainty in consuming genetically modified foods. PR3: Compared to other alternatives, genetically modified foods are riskier.
Phobia of New Food Products (PHOB): Adapted from [57], following the recommendations of [59]. PHOB1: I never try new foods. PHOB2: I do not trust foods produced with genetic modification techniques. PHOB3: If I do not know what food is or how it has been produced, I will not try it. PHOB4: I don’t like foods from other countries. PHOB5: I am afraid of foods produced with genetic modification technologies. PHOB6: I am very picky about new foods I try.
Knowledge of This Type of Products (KNOWL): Adapted from [100] KNOWL1: I am familiar with genetically modified foods. KNOWL2: I have a clear understanding of the characteristics of genetically modified foods. KNOWL3: I know what genetically modified foods are. KNOWL4: I know more about genetically modified foods than other people.
Subjective Norm (SN): Adapted from [97] SN1: People who are important to me think I should eat genetically modified foods. SN2: People who influence me think I should eat genetically modified foods. SN3: People whose opinions I value would prefer to eat genetically modified foods.

### 3.3. Data Analysis

The response to RO1 and RO2 was determined through the successive application of structural equation modeling with partial least squares (PLS-SEM), followed by importance–performance map analysis (IPMA).

The use of PLS-SEM is particularly recommended when there are doubts about the normality of the latent variables involved in the analysis and when the predictive capacity of the proposed model is also evaluated [101]. This explains why it has been used in studies on the acceptance of new food practices, such as, for example, circular food consumption [102]. The model was adjusted following the protocol [101], which involves assessing the internal scale consistency, convergent validity, and discriminant validity of the scales used to measure the latent variables. Subsequently, the model was adjusted by running partial least squares with percentile bootstrapping and 10,000 sub-samples. This adjustment will allow us to test the hypotheses proposed in the previous section, evaluate the overall model, and determine the effect size of the impact of the explanatory variables on the acceptance of GMFs. Finally, the predictive capacity of the model was evaluated using Stone and Geisser’s Q^2^ and conducting a cross-validated predictive ability test (CVPAT), as described in [103].

Subsequently, we implemented an importance–performance map analysis (IPMA) based on the values of the adjusted path coefficients in PLS-SEM and excluded the control variables. Following [104], the importance of explanatory constructs is quantified by the value of the path coefficient in IU when the relationship between these constructs and IU is predictably positive (PB, KNOWL, and SN). In this case, the importance of construct X is calculated as ${Imp}_{X}=\beta_{X}$. In the case where the path coefficient is negative (PR and PHOB), importance is quantified from the opposite constructs, PR* = −PR for PR and PHOB* = −PHOB for PHOB. Thus, the value of importance is the absolute value of the path in such a way that if the path for construct X, with a negative relation with IU, is β_X_ < 0, the importance of X* is ${Imp}_{X^{*}}=\left|\beta_{X}\right|$.

In the case of variables with a positive relationship to IU, performance was quantified based on the average of the items weighted by their loadings, normalized to a scale of 100. In our case, this normalization is achieved by multiplying this average by 10 because the scales are referenced from 0 to 10. On the other hand, for variables with a negative relationship with IU, performance is calculated as that of the complement of X, X* = −X. That is, if the performance for X is ${Per}_{X}$, for X*, it is calculated as ${Per}_{X^{*}}=$ 100 − ${Per}_{X}$.

**Figure 2:**
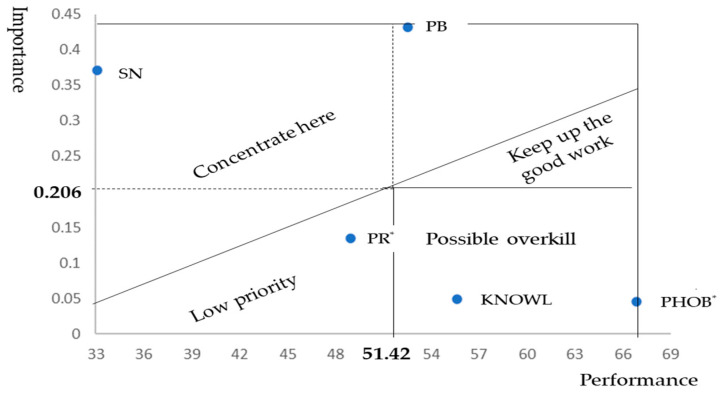
Importance–performance map analysis schema (adapted from [34]).

In this work, the presentation of the importance–performance map differs from that of the conventional map, which divides the two-dimensional plane into four equal quadrants, as also used in [104]. Instead, we use the approach of [34], as it provides greater discriminatory power than conventional interpretation. Figure 2 shows how by following [34], the IPM is interpreted.

## 4. Results

### 4.1. Descriptive Statistics and Measurement Model Analysis

Table 3 presents the descriptive statistics of the items corresponding to the latent variables in the model, as shown in Figure 1. Regarding the response variable IU, the items were slightly above 5, indicating a certain tendency toward acceptance. Additionally, IU2 was significantly higher than 5.

Concerning the attitudinal variables, while the items related to PB were generally significantly above 5, those related to PR varied; some were significantly below 5 (perception of danger), while others were above (perception of uncertainty). Among the perceived control items, the PHOB scores were clearly below the neutral value of 5. Furthermore, in the KNOWL items, mainstream perception significantly leans toward considering oneself knowledgeable, as three out of the four items have values above 5. Finally, all SN items showed a significant tendency toward negative perception, as they were slightly above 3.

Table 3 also demonstrates that the scales exhibit internal consistency, as all constructs have a Cronbach’s alpha and composite reliability greater than 0.7 but less than 0.95. Additionally, the scales generally showed good convergent reliability because the average variance extracted (AVE) was greater than 0.5, and most factor loadings exceeded 0.7. The exceptions were PHOB3 and PHOB4 within the PHOB variable. However, in these cases, the factor loadings were still clearly above 0.6 and very close to 0.7. Moreover, their significance level was below 1%, and the other measures of internal consistency and convergent reliability remained satisfactory.

Table 4 shows that the scales have a clear discriminant validity. They meet the Fornell–Larcker criterion, as the Pearson correlations are lower than the square root of the average variance extracted, and the heterotrait–monotrait ratios between the variables are below 0.85 [101].

### 4.2. Structural Model Analysis

Table 5 presents the model fit results shown in Figure 1. According to [101], the overall fit is high, with *R*^2^ = 70.7%, as indicated in Table 2. The model does not exhibit collinearity issues, as all variables have a variance inflation factor (VIF) below 3.3. Attitudinal variables significantly influence IU, with PB showing a path coefficient (β) of 0.431 and a *p*-value (*p*) < 0.001 and PR having β = −0.134 (*p* < 0.001). Regarding perceived control variables, both PHOB (β = −0.046, *p* = 0.041) and KNOWL (β = 0.049, *p* = 0.032) are significant, while for SN, β = −0.134 (*p* < 0.001). Among sociodemographic variables, only age is significant, with younger individuals showing a greater predisposition to consume GMF (β = −0.115, *p* = 0.011). Additionally, for PB and SN, the effect size of the relationship between exogenous variables and IU is moderate. For the other variables, that size effect is small.

Table 6 presents the results of the predictive capacity analysis for the proposed model. Following [101], the model can be considered predictive, because Q^2^ > 0. Additionally, the CVPAT analysis shows that the proposed model significantly outperforms the benchmark indicator average but not the parsimonious linear model. In the latter case, although the linear model performs better than the one proposed in this study, this improvement is not statistically significant. Therefore, we can conclude that the proposed model has good but not excellent predictive capacity [103].

### 4.3. Importance–Perfomance Map-Analysis

Table 7 presents the importance and performance values used to construct the IPM and derives conclusions from the analysis. The importance of PB, KNOWL, and SN corresponds to the path coefficients from Table 5, while their performance is calculated as the average of the items composing these constructs, weighted by their factor extractions, and expressed on a scale of 100. For PHOB and PR, negations were analyzed. Therefore, PHOB* = −PHOB can be interpreted as the absence of food neophobia, and PR* = −PR, which can be understood as the perception of safety. Because the path coefficient of PR is −0.134, the importance of its opposite, PR*, is ${Imp}_{{PR}^{*}}=$ 0.134. Given that the performance of PR is ${Per}_{PR}=$ 51.017, the performance of PR* is ${Per}_{PR*}=$ 100 − 51.017 = 48.983. Analogously, because the path coefficient of PHOB is β = −0.046, the importance of PHOB* and ${Imp}_{{PHOB}^{*}}=$ 0.046. Similarly, given that ${Per}_{PHOB}$ = 33.116, the performance of PHOB* is ${Per}_{PHOB*}=$ 100 − 33.116 = 66.884.

**Figure 3:**
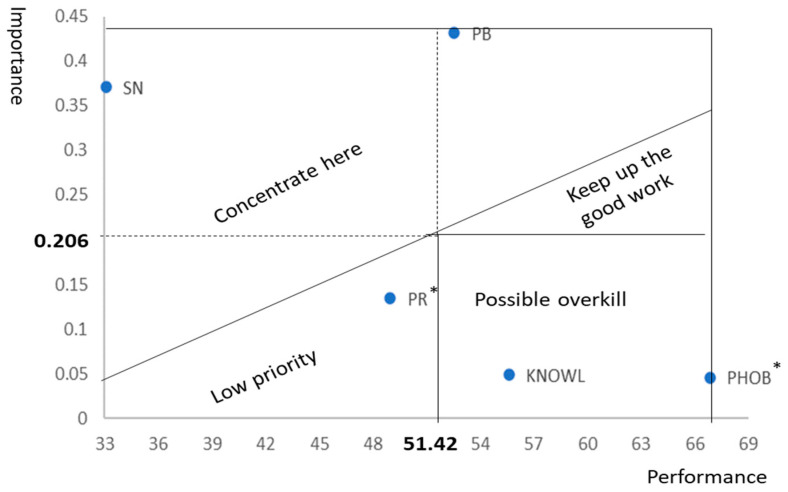
Importance performance map.

The IPM shown in Figure 3 clearly indicates that special attention should be paid to perceived benefits and subjective norms. Both public authorities and firms can influence these constructs through measures, such as changes in labeling policies [16] and public education on biotechnology [105]. By contrast, constructs related to control fall within the overkill zone and perceived risk do not need special attention.

## 5. Discussion

### 5.1. General Overview

This study had two research objectives (ROs). The first (RO1) analyzed the adequacy of a TPB-based model to explain the intention to use (IU) genetically modified food (GMF). This model was fitted using PLS-SEM on a sample of Spanish consumers for 2023. The results indicated that the model fit was good, with an R^2^ of 70% [101]. This adequacy is reinforced by its predictive capacity, given that Stone–Geisser’s Q^2^ > 0 and, in the cross-validated predictive ability test [103], the proposed model significantly outperforms the benchmark referred to as the “average indicator”.

All proposed explanatory variables—perceived benefits (PB), food neophobia (PHOB), perceived risk (PR), knowledge (KNOWL), and subjective norms (SN)—significantly influence IU with the expected sign, meaning that the path coefficients of PB, KNOWL, and SN are positive, while those of PHOB and PR are negative.

In RO2, which aimed to determine which variables should be given special attention to ensure the successful adoption of GMF, we found that the variables requiring performance improvement were perceived benefits and social norms.

Regarding attitude-related variables, the results align with mainstream findings in the literature on GMF acceptance. The significant impact of PB on GMF acceptance has been widely documented [22,44,45,46,47,48,49,50]. It is consistent with the findings obtained before the emergence of modern gene-editing techniques in Spain [22] and the USA [45]. It is also in line with reports following the advent of more advanced techniques in the mid-2010s, in cultural and regulatory contexts as diverse as Asian countries like Taiwan [44], China [47,49], and Japan [48]; European countries like Italy [1]; and Central American regions such as Costa Rica [46] and Panama [46].

Similarly, a statistically significant negative relationship of perceived risk has been well established in the GMF context across various continents and cultures. This includes European countries such as Spain [22] and the Czech Republic [55]; North American countries such as the United States [45] and Canada [53]; Central and South-American states such as Costa Rica [46] and Brazil [106], Asian countries [44,48]; or African countries such as Ghana [54].

Likewise, our findings regarding the influence of perceived control variables on IU are consistent with existing literature. The relevance, albeit with a small effect size, of the significant negative impact of PHOB in explaining IU aligns with reports in other countries such as China [37,64] and the United States [64].

Furthermore, the positive relationship between KNOWL and IU is supported by previous studies on GMF acceptance [36,37,45,61,72]. These findings encompass North America [37,45,72], Arab countries such as Jordan [36], and European countries such as the United Kingdom, France [61], and Italy [1,61].

The importance of SN in explaining GMF acceptance must be understood within its traditional role in food consumption decision-making studies across several cultural settings [32]. People tend to follow social norms in their food choices, as these norms provide key information about the safety, nutritional value, and appropriateness of certain foods or diets [72]. In the context of GMF, our results align with those obtained in European countries such as Italy [17,27], and Iran [28].

Regarding sociodemographic variables, while the predominant literature suggests that women are generally more reluctant to try novel foods, some studies, including this one, do not find a significant impact in this regard in culturally diverse countries such as China [47], Iran [89], and Vietnam [90], as well as in Spain.

Additionally, the negative relationship between age and usage intention aligns with the literature, which generally indicates that younger individuals are more accepting of food innovations in culturally diverse contexts such as the Middle East and North African region [35] or Italy [102].

The IPMA findings on the key role of PB and SN in driving GMF consumption are consistent with those of previous studies that have identified these factors as barriers to GMF expansion. On one hand, widespread skepticism toward GMF has often been attributed to a lack of perceived benefits compared to conventional foods [7,41,59,72,81]. On the other hand, the low performance of the subjective norm construct in Spain (37 out of 100) is consistent with other studies indicating weak consumer adherence to GMF, with an acceptance rate of only 36% in this country [20]. It is worth noting that SN is often a particularly relevant factor in decision-making when knowledge of the topic is limited [75], as is the case with GMF [20,47,79].

On the other hand, it is also noteworthy that the IPMA indicates that PR and KNOWL are not variables that require special attention for the generalization of GMF use. Regarding PR, the analysis of its items suggests that there is no excessive perception of danger, but rather uncertainty about its long-term effects. This uncertainty should gradually dissipate as the implementation of GMFs progresses without causing health issues, which, in any case, have still not been demonstrated.

Similar observations can be made about KNOWL. The responses given to its items show a value (in some cases, even close to 7) significantly higher than that of SN, which also measures the availability of information for consumers regarding GMF. Furthermore, the path coefficient of SN is greater than that of KNOWL, meaning that an increase in SN has a greater influence on GMF acceptance than an increase in KNOWL. Therefore, it seems logical to prioritize SN over KNOWL, as the former is a strategically more relevant variable, given that its item evaluations are much lower. A favorable SN toward GMF is “easier” to enhance than KNOWL, as SN stands from lower evaluations. Moreover, the marginal impact of SN on IU is greater than that of KNOWL.

### 5.2. Theoretical and Practical Implications

We have shown that a TPB-based model provides a conceptual framework that allows for a comprehensive understanding of the drivers of willingness to consume GMF. The presented model explains approximately 70% of the variability in intention to use, so we can admit that it possesses an acceptable predictive capacity.

The application of a mix of analytical tools, such as PLS-SEM and IPM, adds to the evaluation of the model’s predictive capacity, with a prognosis on which of the factors considered relevant should receive special attention [104]. To the best of our knowledge, no similar analysis has been conducted in the field of analyzing acceptance of novel food technologies.

The results of this study have practical implications that may be useful for producers and regulators to promote the use of GMF by humans, in the geographical context of this study, which is a country in the European Union. These recommendations should obviously aim to increase the perception of benefits or, alternatively, the perception that the subjective norm is favorable to the use of GMFs.

Regarding PB, the possibility of increasing productivity can allow producers to compete on prices. In fact, GMF has been shown to be a useful tool to combat food scarcity in regions facing poverty issues [16]. So, some practical issues in this regard are:

- Many consumers would not completely reject GMF but would be willing to consume it in exchange for a price reduction [13,17,107,108]. Although GMF is not perceived as risky, foods made using traditional methods are less attractive [63]. Thus, producers should strive to offer food products with the same qualities as their non-genetically modified counterparts (taste, appearance) but at a lower price.
- As for competing on price, it is remarkable that the European Union experienced high inflation in food products between 2022 and 2023 [109], so the existence of more affordable products in this context may gain interest. These contexts should be considered especially favorable for introducing GMF into the market, as they are the ones in which potential consumers appreciate discounted prices.

The ability to endow GMF with special qualities allows for the creation of more personalized products and makes them suitable to be used in market segmentation strategies [31]. This allows us to make the following considerations:

- GMF can be used as a tool to compete for specific consumer segments. For example, in those interested in sustainability and ecology, or those seeking special nutritional characteristics [1,41]. Since these benefits, unlike price, are not visible, they should be communicated through labeling that clarifies their benefits, such as “grown without pesticides” or “with 25% more vitamin C”. This measure could shift the narrative from “potential risk” to “real benefit” [81].
- GMF can also provide conventional foods with a better aesthetic appearance [59], which may increase their acceptance as a better aesthetic appearance is often associated with better taste [110].

Acting on subjective norms involves improving the social perception of GMF. Some measures to achieve this include:

- Creating accessible public platforms where the development of GMFs, their benefits, and the rigorous safety controls they undergo are explained clearly and scientifically.
- These platforms could be supported by prominent figures in the culinary field and overseen by independent organizations, such as universities and research centers.
- The projection of GMF can be strengthened through altruistic initiatives such as donating food to regions affected by food insecurity or demonstrating how climate-resistant seeds benefit local farmers. It is crucial to communicate that GMF is part of the solution to global problems such as climate change, food scarcity, and soil degradation.
- Workshops, talks, and practical demonstrations could be organized in communities, schools, and agricultural fairs to educate students about the genetic modification process and its advantages.

These actions would not only improve public perception, but also foster a more informed and balanced understanding of GMF.

The perception of the benefits of GMF and the improvement of its perception are also mediated by the regulation of labeling for GMF in the European Union, which at the moment of developing this study is strict, discriminatory, and unfair [1,5,15]. In many countries, such as the United States, Canada, or Brazil, the production and/or labeling regulations are based on product characteristics, while in the European Union, they are regulated based on the process; that is, how it was made [20]. This has led to the cultivation and consumption of GMF being more widespread in the Americas than in Europe [110]. This generates paradoxes in Europe. For example, a conventional crop that has not used pesticides can be labeled as “organic”, a designation highly valued by consumers, while a GMF that does not require pesticides due to its design must carry the label “genetically modified”, without the possibility of adding the “organic” label [15].

Chinese regulatory framework occupies an intermediate position between the strict labeling regulations of Europe and the more lenient regulations concerning information about the origin of GMOs in North America. The country has adopted a balanced approach by advocating for the use of modern technologies, such as genetic modification and genome editing, to promote a more productive and sustainable agricultural sector. Despite that Chinese society appreciates natural food [49], Chinese citizens also actively engage in discussions regarding the benefits and drawbacks of GMFs [111].

In this context, although GMO production in China is lower than in American countries, it is significantly higher than in European Union nations [111]. Consequently, greater acceptance of GMF in the European Union would require consideration of labeling and production regulation issues, such as:

- GMF labeling should be regulated under the principles of legal certainty, non-discrimination, proportionality, and scientific adaptability. For example, this principle would imply the possibility of considering a GMO that does not require pesticides in its production as organic.
- A distinction can be made between products that introduce new genes and those that only have genes edited using CRISPR techniques [5], as the latter has a better perception among informed consumers [51].
- Further expanding on the previous consideration, several instances have pointed out that labeling foods as “genetic modified” derived from organisms to which no external genes have been inserted could even be avoided [15].

### 5.3. Limitations of This Study

This study has some limitations. Firstly, the sample used in this paper was conducted with citizens from a specific European country, Spain. It is important to highlight that the cultural and historical aspects are often relevant for understanding the intention to consume GMF [53,71]. Such differences are evident between different continents [7,20,37,82] and within the European Union [20,31]. Consequently, the conclusions drawn in this study should be extrapolated carefully to other countries, regardless of whether they are within the European Union. For example, while the acceptance and use of GMF are higher in the United States than in the European Union [82], perceptions also differ between European countries, including Spain [20,51,61].

Note that this study measures the consumer’s subjective knowledge about GMF, which, although often correlated with objective knowledge [68], is not exactly the same [69]. Thus, incorporating this second type of knowledge could yield different results, both in the importance of this variable in the formation of IU and in its strategic role when considering this variable in an IPMA.

However, it should be emphasized that this second type of knowledge is more difficult to measure, as it requires examining the respondents’ knowledge rather than simply asking for their opinion. The fact that the respondent may feel they are being evaluated could lead to a significant proportion of individuals who would otherwise be willing to participate in an opinion-based survey becoming reluctant to take part if part of it involves judging their background on GMF.

Furthermore, the implications of this research may not be broadly applicable over the medium- and long-term because of the significant challenges associated with the regulatory aspects of GMF [15] and the continuous evolution of biotechnology techniques [40,112]. In fact, consumers express higher acceptance of GMF when informed about newer genetic modification techniques, such as gene editing, and their differences from older methods such as transgenesis [21,78]. Therefore, the perception of GMF acceptance requires conducting longitudinal studies and analyses throughout the evolving process of biotechnology techniques applied to food.

## 6. Conclusions

This study examined perceptions in a sample from Spain regarding willingness to consume GMF. It tests a TPB-based model and performs an IPMA that identifies the factors that truly require special attention for the commercial development of GMF.

Both the model fit and predictive capacity were good; therefore, the analyses based on it were robust. It is observed that all the proposed explanatory variables have a significant influence on the intention to consume GMF. However, while the variables with the greatest impact are perceived benefits and subjective norms, with a moderate effect size, the effect size of the other variables is small. The IPMA identifies that consumers’ perceived benefits and subjective norms are variables that should be targeted to spread the use of GMF. In light of this analysis, several lines of action have been identified concerning market segmentation policies, price competition, and labeling and advertising strategies aimed at enhancing both perceived benefits and influencing the social perception of GMF.

## Figures and Tables

**Table 1:** Sample profile.

Variable	Number	Percentage
Gender		
Male	280	38.46%
Female	380	52.20%
Other Prefer not Answer	68	9.34%
Age		
Less Than 25 Years	183	25.14%
Between 26 and 35 Years	122	16.76%
Between 36 and 45 Years	63	8.65%
Between 46 and 55 Years	128	17.58%
56 Years and More	174	23.90%
Nonanswered	58	7.97%
Academic Degree		
Less Than Secondary Studies	22	3.02%
Secondary	194	26.65%
University	449	61.68%
Nonanswered	63	8.65%
Monthly Income		
Less Than EUR 1000	188	25.82%
Between EUR 1000 and EUR 1749	169	23.21%
Between EUR 1750 and EUR 2499	77	10.58%
Between EUR 2500 and EUR 3000	50	6.87%
More Than EUR 3000	38	5.22%
Nonanswered	206	28.30%

**Table 3:** Descriptive statistics and measures of scale validity.

	Mean	SD	Factor Loading	CA	CR	AVE
Intention to Use (IU)				0.872	0.882	0.886
IU1	5.023	3.202	0.949 ***			
IU2	5.641 ***	3.433	0.934 ***			
Perceived Benefits (PB)				0.942	0.944	0.851
PB1	5.464 ***	3.04	0.921 ***			
PB2	5.298 ***	3.026	0.946 ***			
PB3	5.415 ***	2.911	0.908 ***			
PB4	4.863	2.772	0.914 ***			
Perceived Risk (PR)				0.796	0.850	0.710
PR1	4.449 ***	2.871	0.903 ***			
PR2	6.412 ***	2.926	0.703 ***			
PR3	4.981	2.925	0.907 ***			
Food Neophobia (PHOB)				0.831	0.924	0.532
PHOB1	3.025 ***	3.045	0.743 ***			
PHOB2	3.033 ***	2.839	0.841 ***			
PHOB3	4.567 ***	3.393	0.661 ***			
PHOB4	1.997 ***	2.619	0.659 ***			
PHOB5	2.643 ***	2.793	0.737 ***			
PHOB6	4.35 ***	3.059	0.721 ***			
Knowledge (KNOWL)				0.898	0.912	0.764
KNOWL1	7.223 ***	2.794	0.833 ***			
KNOWL2	5.543 ***	3.082	0.900 ***			
KNOWL3	5.613 ***	3.011	0.917 ***			
KNOWL4	4.301 ***	3.05	0.845 ***			
Social Norm (SN)				0.946	0.946	0.903
SN1	3.36 ***	2.867	0.947 ***			
SN2	3.354 ***	2.868	0.954 ***			
SN3	3.203 ***	2.910	0.950 ***			

Note: SD = standard deviation, CA = Cronbach’s alpha, CR = composite reliability and AVE = Average variance extracted, and “***” significantly different from 0 at a 1% significance level.

**Table 4:** Matrix of discriminant validity analysis.

	IU	PB	PHOB	PR	KNOWL	SN	Gender	Age
IU	**0.941**	*0.842*	*0.646*	*0.24*	*0.351*	*0.789*	*0.072*	*0.187*
PB	0.766	**0.923**	*0.595*	*0.175*	*0.307*	*0.663*	*0.073*	*0.165*
PHOB	−0.556	−0.542	**0.843**	*0.401*	*0.164*	*0.485*	*0.158*	*0.157*
PR	−0.236	−0.184	0.355	**0.730**	*0.133*	*0.112*	*0.037*	*0.266*
KNOWL	0.318	0.29	−0.152	−0.111	**0.874**	*0.319*	*0.039*	*0.107*
SN	0.72	0.626	−0.428	−0.119	0.303	**0.950**	*0.093*	*0.048*
Gender	−0.067	−0.071	0.127	0.023	0.003	−0.090	**1.000**	*0.052*
Age	−0.174	−0.160	0.142	0.248	−0.106	−0.047	0.052	**1.000**

Note: In the principal diagonal (bolded numbers), the squared average variance is extracted. Above the principal diagonal (italics) are heterotrait–monotrait ratios. Pearson’s correlation was obtained above the principal diagonal.

**Table 5:** Path coefficients of the model proposed in Figure 1.

Path	β	t-Ratio	*p* Value	VIF	f^2^	Decision on Hypothesis
PB -> IU	0.431	12.143	<0.001	2.002	0.316	H1(+): Acceptance
PR -> IU	−0.134	4.81	<0.001	1.617	0.038	H2(−): Acceptance
PHOB -> IU	−0.046	2.045	0.041	1.206	0.006	H4(−): Acceptance
KNOWL -> IU	0.049	2.144	0.032	1.135	0.007	H3(+): Acceptance
SN -> IU	0.371	11.603	<0.001	1.745	0.269	H5(+): Acceptance
Gender -> IU	0.037	0.877	0.381	1.094	0.009	
Age -> IU	−0.115	2.531	0.011	1.022	0.001	

Note: The determination coefficient is 70.7%; β the path coefficient, VIF the variance inflation factor, and f^2^ size factor.

**Table 6:** Measures of prediction capability of the assessed model.

	Measures of PLS-SEM Predict			CVPAT (Benchmark: Indicator Average Value)			CVPAT (Benchmark: Parsimonious Linear Model)		
Construct	Q^2^	RMSE	MAE	ALD	t-Ratio	*p* Value	ALD	t-Ratio	*p* Value
IU	0.691	0.558	0.439	−6.659	18.155	<0.001	0.08	1.2	0.23

Notes: ALD is the average loss difference between the average loss of the model in Figure 1 and the benchmark model (indicator average value and parsimonious linear model).

**Table 7:** Values used to construct IPM in Figure 3.

Factor	Importance	Performance
PB	0.431	52.529
PHOB*	0.046	66.884
PR*	0.134	48.983
KNOWL	0.049	55.634
SN	0.371	33.069
Max	0.431	66.884
Min	0.046	33.069
Average	0.206	51.420

Note: PHOB* represents the opposite constraint of PHOB and PR* is the opposite construct of PR.

## Data Availability

The raw data supporting the conclusions of this article will be made available by the authors on request.

## Abbreviations

The following abbreviations are used in this manuscript:

GMF	Genetic modified food
GMO	Genetic modified organism
TPB	Theory of Planned Behavior
PLS-SEM	Partial least squares–structural equation analysis
IPM	Importance–performance map
IPMA	Importance–performance map analysis
PB	Perceived benefit
PR	Perceived risk
PHOB	Food neophobia
KNOWL	Knowledge
SN	Subjective norm
CVPAT	Cross-validated predictive ability test
VIF	Variance inflation factor
f^2^	Effect size
R^2^	Determination coefficient
Q^2^	Stone–Geissers’ Q^2^
